# Papillary thyroid microcarcinoma and papillary thyroid carcinoma: Clinical characteristics and stratification of treatment strategies

**DOI:** 10.1371/journal.pone.0327423

**Published:** 2025-07-09

**Authors:** Congcong Li, Qiang Li, Xiao Shi, Shuang Han, Xiao Song, Xueqian Li, Xuewei Zhuang

**Affiliations:** 1 Department of Clinical Laboratory, Shizhong District People’s Hospital of Jinan, Jinan, Shandong, China; 2 Department of Surgery, Shizhong District People’s Hospital of Jinan, Jinan, Shandong, China; 3 The Affiliated Taian City Central Hospital of Qingdao University, Jinan, Shandong, China; 4 Department of Clinical Laboratory, Shandong Provincial Third Hospital, Shandong University, Jinan, Shandong, China; Università degli Studi del Piemonte Orientale Amedeo Avogadro: Universita degli Studi del Piemonte Orientale Amedeo Avogadro, ITALY

## Abstract

**Aim:**

Exploring the clinical differences between papillary thyroid micarcinoma (PTMC) and papillary thyroid carcinoma (PTC), optimizing clinical decision-making pathways, and reducing excessive medical behavior while ensuring therapeutic efficacy.

**Method:**

Patients diagnosed with PTMC or PTC by pathological histology from May 2023 to May 2024 at Jinan Shizhong District People’s Hospital were retrospectively analyzed. PTMC refers to thyroid papillary carcinoma with a maximum diameter of ≤1 cm.

**Results:**

There were 186 patients (PTMC group) whose maximum tumor diameter was ≤ 1 cm and 45 patients (PTC group) whose maximum tumor diameter was > 1 cm. The patient’s age was (45.97 ± 10.63) years for the PTMC group and (45.31 ± 11.55) years for the PTC group. No statistically significant differences existed between the two groups in sex, age, BRAF V600E gene mutation, tumor multifocality, and capsular invasion (*P* > 0.05). Between the two groups, there were statistically significant (*P* < 0.05) differences in TNM staging, the thyroid imaging reporting and data system (TI-RADS) staging, and cervical lymph node metastasis.

**Conclusions:**

Thyroid surgery, thermal ablation, and active monitoring are different approaches in the stratified treatment of PTMC and PTC. To avoid overtreatment and improve the quality of life of the patients, personalized treatment plans should be developed according to the test results of TNM stage, TI-RADS classification, and cervical lymph node metastasis.

## 1. Introduction

Papillary thyroid carcinoma (PTC), which originates in the follicular cells of the thyroid gland, is a common pathological type of thyroid cancer (TC). It is a low-grade malignant tumor with excellent prognosis, and the 5-year survival rate is > 95% [1]. Papillary thyroid microcarcinoma (PTMC) is a subtype of PTC. In general, the TC is referred to as PTC if the maximum tumor size is > 1 cm and PTMC when the maximum tumor size is ≤ 1 cm.

It has been reported that PTMC accounts for about >50% of the PTC cases [2].Both PTMC and PTC have similar histomorphology and malignancy, as the cancer cells of PTMC and PTC have the same nuclear features and papillary arrangements, and they are both slow growing tumors that are not very aggressive. Nevertheless, they still have some key differences. While PTC is usually found when the patients realize that there are lumps in their neck, PTMC is an early-stage disease with no obvious clinical symptoms and is often discovered during physical examination. It has been reported about 5%–10% of the PTMC may grow or spread to nearby lymph nodes within 10 years [3].Compared to elderly patients, young PTMC patients (under 40 years old) have a slightly higher risk of progression [4–6]. Tumor growth can occur to PTMC patients during pregnancy, and a small number of cases show biologically aggressive features (e.g., early metastasis and lymph node involvement) [7]. It has been reported that 44.5% of the patients have factors that contribute to tumor invasiveness [8]. The invasive tumors increase the difficulty of treatment and decrease the patient’s quality of life or survival. Good prognosis relies on both early treatment and choosing the appropriate treatment scheme.

There are currently three main treatment methods for PTC and PTMC, i.e., surgical resection, thermal ablation, and active monitoring. Surgical resection is the most applied method that eliminates carcinoma thoroughly with lymph node clearance, but it leaves a large surgical wound and requires lifelong medication after the surgery. The patient is also at risk of complications such as damaging the parathyroid gland and the recurrent laryngeal nerve. Thermal ablation is a popular minimally invasive treatment that reduces the surgical wound and partially preserves the thyroid function. It has fewer complications than surgical resection, but the patient is at risk of recurrence because of residual cancer cells. Active monitoring avoids the complications caused by thyroidectomy, but patients need to regularly have ultrasound examinations, and it can be psychologically taxing for patients to knowingly live with unresected cancer. With the development of medical technology, many healthcare workers have realized that the overtreatment of PTMC tends to take place in current clinical settings and have advocated for personalized treatment plans based on comprehensive evaluations [9,10].

The purpose of this study is to analyze the differences in clinical characteristics between PTMC and PTC, and on the premise of ensuring the treatment effect, prevent the waste of medical resources and avoid patients being overtreated. It aims to improve the overall level of medical services and ensure that patients can receive the best treatment plan.

## 2. Methodology

This study was approved by the ethics committee of Jinan Shizhong District People’s Hospital (No. SYLL-2023–0001). Patients diagnosed with PTC or PTMC at Jinan Shizhong District People’s Hospital from May 2023 to May 2024 based on surgical pathology were reviewed. The patient data included age, sex, tumor location, TNM staging, thyroid imaging reporting and data system (TI-RADS) classification, maximum tumor diameter, BRAF V600E mutation, capsular invasion, cervical lymph node metastasis, and tumor multifocality. Patients were included if they (1) were at least 18 years old and had complete clinical data (2) received thyroid surgery for the first time without prior treatment (3) received an ultrasound examination before the surgery (4) did not have other tumors or complications. Patients were excluded if they had recurrent PTMC or PTC, or had other thyroid diseases such as nodular goiter, thyroid lymphoma, Graves’ disease, etc.

According to “Chinese Expert Consensus on Diagnosis and Treatment of Papillary Thyroid Microcarcinoma”, which uses World Health Organization (WHO) criterion, PTMC was defined as PTC with a maximum tumor diameter of 1 cm. The TNM staging was created based on the AJCC Cancer Staging Manual (8^th^ edition) [11–13]. The TI-RADS score was calculated based on the Thyroid Imaging Reporting and Data System guideline from the American College of Radiology (ACR TI-RADS) [14]. The cell tissues obtained by ultrasound-guided fine-needle aspiration biopsy were used for the BRAF V660E gene testing [15].

Data were analyzed using SPSS 27.0. Quantitative data were expressed as mean ± standard deviation (mean ± SD), and their differences were evaluated using the two-sample t-test if the data had a normal distribution or the Mann–Whitney U test if otherwise. Count data were expressed as cases and ratios (n, %), and the inter-group comparison was performed using the χ^2^ test. The relationship between the various clinical characteristics was studied using the univariate analysis method [16]. The correlation analysis between one-way ordinal categorical variables was performed using the Kruskal–Wallis test. Differences were considered statistically significant when* *P* < 0.05;** *P* < 0.01.

## 3. Results

### 3.1. Basic information

A total of 231 patients were included in this study. Their age ranged in 21–74 years, and the average age was (45.84 ± 10.79) years. There were 45 patients in the PTC group, and their age was (45.31 ± 11.55) years. The group included 11 male patients (24.4%) and 34 female patients (75.6%). There were 186 patients in the PTMC group, and their age was (45.97 ± 10.63) years. The group included 44 male patients (23.7%) and 142 female patients (76.3%). In the PTC group, 17 patients (37.8%) did not have cervical lymph node metastasis, and 28 patients (62.2%) had cervical lymph node metastasis. In the PTMC group, 121 patients (65.1%) did not have cervical lymph node metastasis, and 65 patients (34.9%) had cervical lymph node metastasis. For each group, the TI-RADS classification was as follows: PTC group, Class 4A, 5 (11.1%); Class 4B, 1 (2.2%); Class 4C, 12 (26.7%), Class 5, 27 (60.0%); PTMC group, Class 4A, 40 (21.5%); Class 4B, 42 (22.6%); Class 4C, 73 (39.2%), Class 5, 31 (16.7%). The average tumor diameter was (1.51 ± 0.46) cm in the PTC group and (0.59 ± 0.23) cm in the PTMC group. Significant differences existed between the two groups in TI-RADS classification, TNM stage, and cervical lymph node metastasis (*P* < 0.05). No statistically significantly differences existed between the two groups in the tumor location, BRAF V600E mutation, tumor multifocality, and capsular invasion (*P* > 0.05) Table 1.

**Table 1:** Basic information of patients.

	PTMC^†^	PTC^†^	*P*
Number of cases	186	45	
Age years, Range	21–70	26–74	
Age years, Mean ± SD	45.97 ± 10.63	45.31 ± 11.55	0.842
Age, Segment (>40 vs younger)	124/62	31/14	
Sex (M/F)	44/142	11/34	0.911
Location of tumor (left/right/both) %	30.6/38.2/31.2	22.2/31.1/46.7	0.097
TNM stage (T1/T2/T3) %	98.9/0/1.1	86.7/11.1/2.2	**0.001**
TI-RADS classification (4A/4B/4C/5) %	21.5/22.6/39.2/16.7	11.1/2.2/26.7/60.0	**0.001**
Maximum tumor diameter(cm)	1.0	3.0	0.001
BRAFV600E mutation (+/ − /deficiency) %	49.5/7.5/43.0	48.9/4.4/46.7	0.513
Cervical lymph node metastasis (+/−) %	34.9/65.1	62.2/37.8	0.001
Multifocality (+/−) %	43.0/57.0	55.6/44.4	0.13
Capsular invasion (+/−) %	35.0/65.0	62.2/37.8	0.053

PTMC, papillary thyroid microcarcinoma; PTC, papillary thyroid carcinoma. P: P-value.

### 3.2. Analysis by age

In the PTC group, 14 patients (31.2%) were 40 years old or younger, and 31 patients (68.8%) were 41 years old or older. In the PTMC group, 62 patients (33.3%) were 40 years old or younger, and 124 patients (66.7%) were 41 years old or older. The two groups had a significant difference (*P* < 0.05) in the segmentation of patients by age (with the cutoff at 40 years old). The incidence of PTMC gradually increased with age and was the highest in the 51–60 years age bracket, although it was the lowest in the > 61 years age bracket Table 2.

**Table 2:** Distribution of patients across age brackets.

Age bracket	PTMC	PTC	95% CI	*P*
21–30	14 (7.5%)	7 (15.6%)	1.45–1.89	
31–40	48 (25.8%)	7 (15.6%)	1.78–1.96	
Subtotal (≤40)	62 (33.3%)	14 (31.2%)		
41–50	53 (28.5%)	13 (28.8%)	1.74–1.90	
51–60	59 (31.7%)	17 (37.8%)	1.68–1.87	
>60	12 (6.5%)	1 (2.2%)	1.76–2.09	
Subtotal (>40)	124 (66.7%)	31 (68.8%)		
Total	186	45		**0.039**

### 3.3. Analysis of clinical characteristics

#### 3.3.1. PTMC group.

Summarizes the data of PTMC patients based on cervical lymph node metastasis, of the 121 cases (65.1%) that did not have cervical lymph node metastasis, the tumor size was ≤ 0.5 cm for 68 cases (56.2%) and >0.5 cm for 53 cases (43.8%). Of the 65 cases (34.9%) that had cervical lymph node metastasis, the tumor size was ≤ 0.5 cm for 22 cases (33.8%) and >0.5 cm for 43 cases (66.2%). (See Table 3). A significant difference existed between them (*P* < 0.05), thus indicating that the probability of cervical lymph node metastasis was higher when the maximum tumor diameter was larger. Between PTMC patients with and without cervical lymph node metastasis, significant differences (*P* < 0.05) existed in sex, tumor location, TI-RADS classification, and tumor multifocality.

**Table 3:** Clinical characteristics of PTMC and PTC cervical lymph node metastasis.

Clinical characteristics	PTMC			PTC		
	Cervical lymph node metastasis		*P*	Cervical lymph node metastasis		*P*
	+	−		+	−	
Number of cases	65	121		28	17	
Tumor size			**0.002**			0.457
≤0.5 cm	22 (33.8%)	68 (56.2%)		25 (89.3%)	16(94.1%)	
>0.5 cm	43 (66.2%)	53 (43.8%)		3 (10.7%)	1 (5.9%)	
Sex (M/F)			**0.006**			0.127
M	23 (35.4%)	21 (17.4%)		9 (32.1%)	2 (11.8%)	
F	42 (64.6%)	100 (82.6%)		19 (67.9%)	15 (88.2%)	
Age			0.167			0.541
21–30	9 (13.8%)	5 (4.1%)		4 (14.3%)	3 (17.6%)	
31–40	15 (23.1%)	33 (27.3%)		4 (14.3%)	3 (17.6%)	
41–50	16 (24.6%)	37 (30.6%)		8 (28.5%)	5 (29.5%)	
51–60	20 (30.8%)	39 (32.2%)		11 (39.3%)	6 (35.3%)	
61–80	5 (7.70%)	7 (5.8%)		1(3.6%)	0 (0%)	
Location of tumor			**0.004**			1.0
Right	26 (40.0%)	45 (37.2%)		7 (25.0%)	7 (41.2%)	
Left	12 (18.5%)	45 (37.2%)		7 (25.0%)	3 (17.6)	
Both	27 (41.5%)	31 (25.6%)		14 (50.0%)	7 (41.2%)	
TNM stage			**<0.001**			**<0.001**
T1aN0MX	0 (0.0%)	119 (98.3%)		0 (0.0%)	1 (5.9%)	
T1aN1aMX	60 (92.3%)	1 (0.85%)		3 (10.7%)	0 (0%)	
T1aN1bMX	5 (7.7%)	0 (0.0%)		1 (3.6%)	15 (88.2%)	
T1bN1aMX	0 (0.0%)	0 (0.0%)		19 (67.9%)	0 (0%)	
T2N0MX	0 (0.0%)	0 (0.0%)		2 (7.1%)	0 (0%)	
T2bN1bMX	0 (0.0%)	0 (0.0%)		2 (7.1%)	0 (0%)	
T3aN0MX	0 (0.0%)	1 (0.85%)		0 (0%)	1 (5.9%)	
T3bN0MX	0 (0.0%)	0 (0%)		1 (3.6%)	0 (0%)	
TI-RADS classification			**<0.001**			0.556
4A	5 (7.7%)	35 (28.9%)		3 (10.7%)	2 (11.8%)	
4B	16 (24.6%)	26 (21.5%)		1(3.6%)	0 (0%)	
4C	28 (43.1%)	45 (37.2%)		6 (21.4%)	6 (35.3%)	
5	16 (24.6%)	15 (12.4%)		18 (64.3%)	9 (52.9%)	
BRAFV600E mutation			0.86			0.709
+	31 (47.7%)	61 (50.4%)		14 (50.0%)	8 (47.0%)	
−	6 (9.20%)	8 (6.60%)		1 (3.6%)	1 (6.0%)	
deficiency	28 (43.1%)	52 (43.0%)		13 (46.4%)	8 (47.0%)	
Multifocality			**0.029**			0.786
+	35 (53.8%)	45 (37.2%)		16 (57.1%)	9 (52.9%)	
−	30 (46.2%)	76 (62.8%)		12 (42.9%)	8 (47.1%)	
Capsular invasion			0.238			0.667
+	16 (24.6%)	21 (17.4%)		10 (35.7%)	5 (29.4%)	
−	49 (75.4%)	100 (82.6%)		18 (64.3%)	12 (70.6%)	

**Fig 1:**
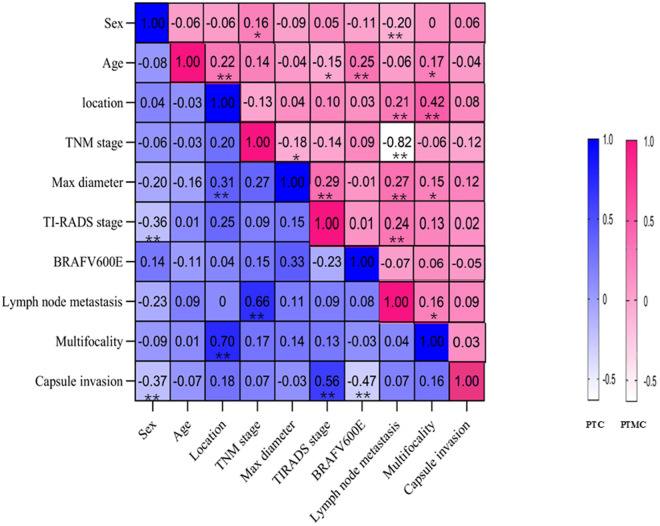
Correlations between clinical characteristics for the PTMC and PTC patients.

The correlation analysis revealed that in the PTMC group, sex was correlated with TNM stage positively and with cervical lymph node metastasis highly significantly negatively. Indeed, male PTMC patients had larger tumor diameter (0.623 ± 0.220 cm vs 0.575 ± 0.228 cm), later TNM stage, and higher TI-RADS score than female patients. In addition, the patient’s age was highly significantly positively correlated with BRAF V600E mutation and tumor location, significantly positively correlated with tumor multifocality, and significantly negatively with the TI-RADS stage. The tumor location was correlated significantly positively with cervical lymph node metastasis and tumor multifocality. A further analysis of the data revealed that multifocal tumors were less likely to occur when the lesion was limited to only the left lobe of the thyroid gland. The TNM stage was correlated significantly negatively with maximum tumor diameter and highly significantly negatively with cervical lymph node metastasis. The maximum tumor diameter was correlated significantly positively with tumor multifocality and highly significantly positively with TI-RADS classification and cervical lymph node metastasis. The cervical lymph node metastasis was correlated significantly positively with tumor multifocality and TI-RADS classification Fig 1. It has been stated in many published research articles that tumor multifocality and high TI-RADS scores are independent risk factors for central cervical lymph node metastasis [17,18], and our findings here agreed well with the literature. Therefore, comprehensive judgment and analysis are needed in carrying out the stratified treatment of PTC and PTMC to reduce the probability of lymph node metastasis.

#### 3.3.2. PTC group.

There were 45 cases in the PTC group, including 11 males (24.4%) and 34 females (75.6%). There were 28 cases (62.2%) with cervical lymph node metastasis and 17 cases (37.8%) without metastasis. There was a significant difference (P < 0.01) in cervical lymph node metastasis between TNM staging and PTC, as shown in Table 3. Sex was correlated with both TI-RADS score and capsular invasion highly significantly negatively. Tumor location was correlated with both maximum tumor diameter and tumor multifocality highly significantly positively. Cervical lymph node metastasis was correlated with TNM stage highly significantly positively. Capsular invasion was also correlated with TI-RADS score highly significantly positively and with BRAF V600E mutation highly significantly negatively. as shown in Fig 1.

## 4. Discussion

Nowadays, thyroid cancer tends to be detected sooner and more frequently, thanks to the rising awareness of physical examinations and the improvement in healthcare services. Delivering the right mode of treatment not only improves the patient’s quality of life but also optimizes the use of healthcare services. The aim of this study is to investigate the clinical and pathological characteristics of PTMC and PTC, identify risk factors, optimize clinical pathways, in order to achieve precise clinical intervention and rational allocation of medical resources.

Between the PTMC and PTC patients, there were statistically significant differences (*P* < 0.05) in the TNM stage, the TI-RADS classification score, and the cervical lymph node metastasis. It has been reported that cervical lymph node metastasis tends to occur in the early stage of PTC and occurs more commonly in the central neck area than the lateral neck area [19,20]. In the PTC group, 62.2% (28/45) of the patients had cervical lymph node metastasis. In the PTMC group, 34.9% (65/186) of the patients had cervical lymph node metastasis, and 68.8% (128/186) of them had a single lesion, most often found in the right lobe of the thyroid. We speculate that due to the right lobe of the thyroid being closer to the common carotid artery and internal carotid artery, the abundant blood supply to the tumor accelerates its growth rate, making it more prone to lymph node metastasis and membrane invasion. The TNM stage was correlated with cervical lymph node metastasis negatively in the PTMC group but positively in the PTC group. The conclusion drawn from the above research is that when the tumor is located in the right lobe of the thyroid gland and the tumor diameter is ≥ 0.5 cm, lymph node metastasis in the central region of the neck is prone to occur. Therefore, the monitoring interval should be appropriately shortened during real-time monitoring to prevent lymph node metastasis, Surgery can be performed to remove the tumor while clearing the lymph nodes.

Among the PTMC patients, male patients were more likely to have cervical lymph node metastasis and had more advanced TNM and TI-RADS stages, likely because men tended to overlook health examinations and treat diseases less promptly. For the PTMC group, age was correlated significantly negatively with TI-RADS classification. Age is considered a risk factor of poor prognosis for PTMC and PTC and the tumor tends to be more aggressive when the patient is younger, likely because young patients have an active metabolism and cells renew at a more rapid rate [21,22].The decrease in the incidence of PTMC and PTC in the > 60 years old age group is likely related to slower cell proliferation in the patients’ body and other aspects of their internal environment. Capsular invasion occurs when the thyroid tumors break through the capsule and invade surrounding blood vessels, organs, and other tissues to cause recurrence or metastasis. There was no statistical difference between the two groups (PTMC and PTC) in capsular invasion.

Over the past decade, expert consensus on the treatment of PTMC has been published in many countries to standardize the treatment of PTMC. In 2015, the American Thyroid Association (ATA) recommended active surveillance for the treatment of PTMC [10]. The “Chinese Expert Consensus on the Diagnosis and Treatment of Papillary Thyroid Microcarcinoma”, which is published in 2016, recommends active monitoring for tumors that are ≤ 0.5 cm in diameter [23].The latest edition of “Expert Consensus on Thermal Ablation of Papillary Thyroid Cancer”, published in April 2024, mentions that thermal ablation is also gradually being adopted in treating PTMC. Regarding PTC, the consensus acknowledges the safety and efficacy of thermal ablation for both T1aN0M0 PTC and T1bN0M0PTC, and it recognizes some preliminary studies on thermal ablation for T2N0M0 PTC. Indeed, research has proven that compared to surgical resection, thermal ablation is no worse regarding efficacy but has much better safety in the treatment of PTC [24]. However, in clinical practice, we still see many patients who dread having cancer and choose surgical resection so that they can eradicate the problem, but total or partial thyroidectomy can be an overtreatment for PTMC patients [10,3]. A lot of clinical studies reported that immediate surgical resection does not provide additional benefit to patients compared to carrying out surgical resection only after thermal ablation or active monitoring [25]. On the contrary, recurrence is less likely when the patient goes through active monitoring first before surgery [26].

Active surveillance is suitable for pregnant women and low-risk PTMC patients with family history and/or small thyroid calcifications [27]. Thermal ablation is preferred if the maximum tumor diameter is > 0.5 cm and the TNM stage is T1bN0M0. Surgical resection is beneficial when there is lymph node metastasis or distant metastasis, or if there is tumor invasion or attachment to nerves or trachea [28]. During active surveillance, patients can switch to surgery if the tumor diameter reaches 13 mm or grows by 3 mm within a short time, or if they have other surgical indications [3,28]. Xu et al. showed that for intermediate-risk thyroid cancer with cervical lymph node metastasis, lobectomy has the same efficacy compared to complete surgical resection [29]. Hence, to partly preserve thyroid function, lobectomy is a feasible alternative to surgical resection. We commend a stratified protocol for the treatment of PTMC based on TNM stage, TI-RADS classification, and cervical lymph node metastasis. (1)Real time monitoring can be performed when TI-RADS is class 4C or 5 [6] and the diameter of the mass is less than 0.5 cm, and there is no cervical lymph node metastasis; (2) When TI-RADS is classified as 4C or 5 and the diameter of the tumor is ≤ 2.0 cm but there is no lymph node metastasis, thermal ablation treatment can be used; (3) TI-RADS is classified as 4C or 5, and if the tumor diameter is greater than 2.0 cm, timely surgical treatment should be performed; (4) Regardless of the diameter of the tumor, immediate surgery and lymph node dissection are required when cervical lymph node metastasis occurs. We also advise physicians to, during their work, publicize the benefits of active monitoring and thermal ablation for PTMC so that the patients can be fully informed, and then provide expert opinions after synthesizing the patient’s own wishes and the examination results.

The strengths and limitations of this study are as follows, By analyzing the different clinical characteristics of PTMC and PTC, we developed a treatment strategy based on risk group stratification and provided our opinion on the ideal treatment of PTMC and PTC. However, the soundness of our conclusions is affected by the limited sample size. In addition, the TI-RADS classification is a subjective measure that depends on the judgment and experience of the physician. To determine robust and reliable treatment strategies for PTMC, future studies should increase the sample size and adopt measures to improve the consistency in assessing patients.

## Supporting information

S1 File Data information. (XLSX)
